# Trends in cardiovascular risk factors among U.S. men and women with and without diabetes, 1988–2014

**DOI:** 10.1186/s12889-017-4921-4

**Published:** 2017-11-22

**Authors:** Xingxing Sun, Tingting Du

**Affiliations:** 1 Department of Anesthesiology, Tongji Hospital, Tongji Medical College, Huazhong University of Science and Technology, Wuhan, 430030 China; 2 Department of Endocrinology, Tongji Hospital, Tongji Medical College, Huazhong University of Science and Technology, Wuhan, Hubei Province 430030 People’s Republic of China

**Keywords:** Diabetes, Cardiovascular risk factors, Trends

## Abstract

**Background:**

Studies evidenced that reduction in cardiovascular disease (CVD) mortality in diabetic patients can be attributed to improvements in major CVD risk factors and evidence-based treatments. Furthermore, studies showed that the relative risk of CVD mortality associated with diabetes compared with non-diabetes is stronger in women than in men. Hence, we aimed to examine trends in CVD risk factors and intervention measures by sex and diabetic status.

**Methods:**

Analysis of 5 distinct cross-sectional National Health and Nutrition Examination Surveys, 1988–1994, 1999–2002, 2003–2006, 2007–2010, and 2010–2014. Since detailed information on nontraditional risk factors such as sleep apnea was not available in each NHANES survey, traditional CVD risk factors including obesity, hypertension, and dyslipidemia were assessed in the study. To assess whether changes throughout the 27-year period differed by diabetes status, a logistic regression analysis was utilized to examine potential interaction effects between survey and diabetes. The similar process was repeated for sex.

**Results:**

Means of all risk factors except body mass index and waist circumference decreased and the prevalence of antihypertensive and lipid-lowering medication use increased over time among diabetic and non-diabetic men and women. For both men and women, survey × diabetes status interaction terms for changes in HDL-cholesterol and triglyceride levels were not statistically significant, while the prevalence of antihypertensive and lipid-lowering medication use increased more in diabetic than in non-diabetic persons (all *P* < 0.001). For women, survey × diabetes status interaction terms indicated that compared with the first survey, total cholesterol, LDL-cholesterol, and non-HDL-cholesterol fallen more in diabetic than in non-diabetic persons (all *P* < 0.001). In the diabetic state, men experienced similar changes in means of all CVD risk factors and the prevalence of antihypertensive and lipid-lowering medication use as women (all *P* for interactions between survey and sex were >0.01).

**Conclusions:**

The major traditional CVD risk factors in diabetic men decreased to the same extent that they did for non-diabetic men. The magnitude of changes in the favorable trends in diabetic women was of similar or greater compared with those among non-diabetic women. Diabetic women had as good an improvement in CVD risk factors as diabetic men.

**Electronic supplementary material:**

The online version of this article (10.1186/s12889-017-4921-4) contains supplementary material, which is available to authorized users.

## Background

A marked decrease in the prevalence of death from cardiovascular disease (CVD) in the United States was observed over the past decades [1]. Although CVD is the leading cause of mortality associated with diabetes, diabetic patients also experienced the decline in CVD mortality [2]. Analyses of consecutive cohorts of the U.S. population from the 1970s through the 1990s found that CVD mortality declined among diabetic men but not among diabetic women [3]. National studies examined mortality trends between 1997 and 2006 showed that CVD death rates among both U.S. men and women with diabetes declined substantially [2]. National surveys conducted early may not accurately reflect the current state of CVD mortality among individuals with diabetes, as many continued advances in treatment approaches have been introduced into contemporary practice. Identifying the underlying factors associated with the decline in CVD mortality is critical for planning future health policy, and prioritizing strategies for primary and secondary prevention. Previous studies have shown that the largest portion of the reduction in CVD mortality can be attributed to improvements in major CVD risk factors and evidence-based treatments [4]. In addition, studies have shown that the relative risk of CVD mortality associated with diabetes compared with non-diabetes is stronger in women than in men [5]. To our knowledge, no national studies have attempted to quantify the trends in certain CVD risk factors and intervention measures among diabetic and non-diabetic men and women in the U.S.. We therefore used data from consecutive nationally representative health surveys spanning 1988 to 2014 to examine the trends in certain major CVD risk factors and intervention measures among diabetic and non-diabetic men and women.

## Methods

### Study population

We used data from 5 consecutive National Health and Nutrition Examination Surveys (NHANES), including 1988–1994, 1999–2002, 2003–2006, 2007–2010, and 2011–2014. The NHANES are a series of cross-sectional health examination surveys. Full details of each survey have been described elsewhere [6, 7]. Briefly, each of the surveys followed a complex stratified, multistage probability cluster design to ensure that the sample is nationally representative of the civilian, noninstitutionalized US population. Participants were interviewed at home for basic sociodemographic and health-related information. After the in-home interview, participants are invited to attend a mobile examination center, where they underwent a set of standardized physical examinations and laboratory measurements. All data were collected according to the standardized NHANES protocols. Each adult participant provided a written informed consent and the NHANES was approved by the National Center for Health Statistics ethics review board.

All participants were asked to complete a standardized questionnaire which provided information on age, sex, race/ethnicity, smoking habits, histories of current and previous illness, and medical treatments during the home interview. There were 17,030, 10,291, 10,020, 12,153, and 11,329 persons aged 20 years or older selected for 1988–1994, 1999–2002, 2003–2006, 2007–2010, and 2011–2014, respectively. We restricted our analyses to non-pregnant adults who completed the examination and with no extreme triglycerides (> 400 mg/dl) and HDL-cholesterol (> 100 mg/dl) values. The remaining 15,310 (89.9%), 9933 (96.5%), 9381 (93.6%), 11,913 (98%), and 11,115 (98.1%) persons in the 5 surveys, respectively, were included in current analysis.

### Measurements

Body mass index (BMI) was calculated as weight (in kilograms) divided by the square of height (in meters). Waist circumference (WC) was measured with a steel measuring tape just above the iliac crest to the nearest 1 mm. Blood pressure (BP) was measured using mercury sphygmomanometers. The last two readings were averaged.

### Biochemical measurements

Fasting plasma glucose (FPG) was measured by the modified hexokinase enzymatic assay (Cobas Mira Chemistry System; Roche Diagnostic Systems, Montclair, NJ). Hemoglobin A1c (HbA1c) was measured with high-performance liquid chromatography and was standardized to the Diabetes Control and Complications Trial. Total cholesterol (TC) and triglycerides were measured enzymatically. HDL-cholesterol was measured by the direct immunoassay method in NHANES 2001–2002 and 2007–2014, whereas it was measured by the heparin manganese precipitation method in NHANES 1988–1994 and 1999–2000. Although there were changes in laboratories, methods, and instruments used for serum lipid measurements across surveys. Lipid measurements from each NHANES were standardized according to the criteria of the Centers for Disease Control and Prevention and the National Heart, Lung, and Blood Institute Lipid Standardization Program. Non-HDL-cholesterol was calculated as TC minus HDL-cholesterol. For persons with triglycerides ≤ 400 mg/dL, LDL-cholesterol was calculated using the Friedewald equation. Laboratory procedures and quality control methods have been described in the NHANES Laboratory/Medical Technologists Procedures Manual (http://www.cdc.gov/nchs/nhanes/nhanes_questionnaires.htm).

### Assessment of CVD risk factors

Nine traditional CVD risk factors were analyzed: BMI, WC, smoking status, BP, TC, triglycerides, HDL-cholesterol, LDL-cholesterol, and non-HDL-cholesterol. These risk factors were chosen because they independently predicted CVD mortality [8–12] and were assessed in all surveys. BP and lipid levels were analyzed regardless of medication use.

### Definitions

According to 2015 American Diabetes Association (ADA) criteria [13], diabetes is defined as an FPG ≥ 126 mg/dl, HbA1c ≥ 6.5% (48 mmol/mol), previously diagnosed diabetes (by self-report), or current use of anti-diabetic medication or insulin.

Persons are classified as current smokers if they reported smoking at least 100 cigarettes in their lifetime and reported smoking now [14].

Systolic/diastolic BP < 130/80 mmHg, and LDL-cholesterol <100 mg/dL are used as the threshold definitions of controlled treatment based on the ADA’s Standards of Medical Care for people with diabetes [15].

### Statistical analysis

Complex survey procedures in SAS 9.2 (SAS Institute) were performed for all analyses. Sample weights were incorporated to produce nationally representative estimates. Continuous variables were expressed as arithmetic means (95% confidence Intervals [CI]) except for triglycerides, which was expressed as geometric means (95% CI) due to its highly skewed distribution. Standard errors of the means (or percentages) used to calculate 95% CI were estimated by Taylor Series Linearization. To maximize the comparability across surveys, all survey data were age-standardized by the direct method to the 2000 US Census population using the age categories of 20–39, 40–59 years, and ≥60 years. The statistical significance of the differences between men and women with or without diabetes were determined using two-way analysis of covariance with gender and diabetes status as the two main effects. Bonferroni correction was applied to adjust *P* values for multiple comparisons. Trends in age-adjusted means of CVD risk factors from 1988 to 1994 to 2011–2014 were assessed using orthogonal polynomial coefficients. To assess if changes in means between the first and last surveys differed by diabetes status or by sex, general linear models were utilized to examine potential interaction effects between survey and diabetes status or between survey and sex. To assess whether changes in the prevalence of CVD risk factors throughout the 27-year period differed by diabetes status, a logistic regression analysis was utilized to examine potential interaction effects between survey and diabetes. Similar processes were repeated for sex. Logistic regression and computed predictive marginals were used to estimate survey trends in age-adjusted prevalence over the 27-year period. A two-tailed *P* value of <0.05 was considered significant.

## Results

Additional file 1: Table S1 listed the characteristics of adults with and without diabetes across the 5 surveys.

From 1988 to 1994 to 2011–2014, statistically significant increasing trends in mean BMI and WC levels were observed among all diabetic and non-diabetic men and women groups (Table 1). For both men and women, absolute increments among diabetic patients were as great as those among non-diabetic persons (for men, *P* for interactions between survey and diabetes status were 0.077 for BMI and 0.027 for WC; for women, *P* for interactions between survey and diabetes status were 0.911 for BMI and 0.061 for WC). In the non-diabetic state, the absolute increments in the mean BMI levels were not significantly different between men and women (test for survey × sex interaction was 0.056), while absolute increments in the mean WC levels were greater for women (7.4 cm) than for men (4.6 cm) (the survey × sex interaction for WC was <0.001). In the diabetic state, absolute increments in the mean BMI and WC levels were comparable for men and women (test for survey × sex interaction were 0.404 and 0.094, respectively).

**Table 1:** Age-adjusted means of cardiovascular risk factors by sex and glucose-defined diabetes status among adults aged 20 years or older, 1988–2014^a^

Risk actors by sex and diabetes status	NHANES 1988–1994	NHANES 1999–2002	NHANES 2003–2006	NHANES 2007–2010	NHANES 2011–2014	*P* Value for Linear Trend, 1988–1994 to 2011-2014^b^
Body mass index (kg/m^2^)						
Non-diabetic Men	26.44 (26.23–26.66)	27.48 (27.25–27.71)	28.07 (27.83–28.31)	28.27 (28.02–28.51)	28.14 (27.90–28.37)	<.0001
Diabetic Men	28.06 (27.35–28.78)	32.43 (30.58–34.28)	31.81 (30.80–32.83)	31.89 (30.94–32.84)	32.69 (31.09–34.29)	<.0001
Non-diabetic Women	26.17 (25.87–26.47)	27.82 (27.45–28.19)	28.00 (27.60–28.40)	28.10 (27.85–28.34)	28.56 (28.21–28.92)	<.0001
Diabetic Women	31.12 (29.59–32.65)	33.50 (31.77–35.24)	34.71 (33.16–36.27)	34.13 (32.32–35.95)	34.83 (33.65–36.00)	<.0001
Waist circumference (cm)						
Non-diabetic Men	95.32 (94.87–95.78)	98.44 (97.86–99.02)	100.18 (99.51–100.85)	100.07 (99.38–100.76)	99.92 (99.31–100.54)	<.0001
Diabetic Men	100.52 (98.49–102.55)	111.72 (107.33–116.12)	109.62 (107.20–112.04)	109.01 (106.11–111.91)	112.42 (108.49–116.36)	<.0001
Non-diabetic Women	87.97 (87.31–88.64)	91.68 (90.74–92.62)	93.31 (92.40–94.22)	93.85 (93.18–94.52)	95.37 (94.60–96.14)	<.0001
Diabetic Women	100.46 (97.22–103.70)	106.74 (103.01–110.47)	108.63 (105.17–112.09)	109.25 (105.63–112.86)	110.24 (107.63–112.86)	<.0001
Systolic blood pressure (mmHg)						
Non-diabetic Men	125.23 (124.57–125.89)	124.33 (123.30–125.35)	124.26 (123.40–125.11)	122.79 (122.25–123.34)	122.59 (121.77–123.41)	<.0001
Diabetic Men	127.91 (125.83–129.99)	128.19 (124.46–131.93)	127.61 (125.54–129.67)	126.13 (124.00–128.26)	126.81 (124.68–128.95)	0.0007
Non-diabetic Women	120.20 (119.62–120.78)	122.11 (121.39–122.83)	120.81 (120.16–121.47)	118.12 (117.42–118.82)	118.64 (117.89–119.39)	<.0001
Diabetic Women	125.31 (123.19–127.44)	130.71 (127.07–134.34)	125.74 (123.32–128.15)	124.66 (122.49–126.84)	123.52 (121.91–125.13)^c^	<.0001
Diastolic blood pressure (mmHg)						
Non-diabetic Men	76.79 (76.37–77.22)	73.88 (73.20–74.56)	71.70 (71.15–72.26)	72.03 (71.31–72.74)	71.86 (71.03–72.69)	<.0001
Diabetic Men	75.83 (74.64–77.02)	75.10 (71.73–78.46)	74.13 (72.06–76.20)	70.51 (67.78–73.25)	72.95 (70.85–75.05)	<.0001
Non-diabetic Women	72.28 (71.83–72.72)	70.84 (70.16–71.52)	69.41 (68.77–70.05)	68.40 (67.51–69.29)	69.67 (68.98–70.36)	<.0001
Diabetic Women	74.16 (73.01–75.31)	69.87 (66.70–73.03)	68.48 (66.80–70.16)	69.42 (67.76–71.08)	69.61 (68.36–70.86)	<.0001
Total cholesterol (mmol/l)						
Non-diabetic Men	5.25 (5.20–5.30)	5.10 (5.04–5.15)	5.15 (5.11–5.19)	5.03 (4.99–5.08)	4.90 (4.86–4.95)	<.0001
Diabetic Men	5.10 (4.92–5.29)	5.09 (4.86–5.31)	5.21 (5.03–5.39)	4.75 (4.59–4.90)	4.73 (4.60–4.87)	<.0001
Non-diabetic Women	5.30 (5.26–5.34)	5.15 (5.10–5.20)	5.27 (5.23–5.31)	5.15 (5.11–5.20)	5.08 (5.04–5.12)	<.0001
Diabetic Women	5.51 (5.38–5.64)	5.32 (5.11–5.53)	5.31 (5.11–5.51)	5.10 (4.96–5.24)	4.92 (4.83–5.02)	<.0001
HDL-cholesterol (mmol/l)						
Non-diabetic Men	1.20 (1.18–1.22)	1.20 (1.19–1.22)	1.27 (1.26–1.29)	1.23 (1.22–1.25)	1.26 (1.24–1.28)	<.0001
Diabetic Men	1.14 (1.10–1.18)	1.07 (1.01–1.14)	1.16 (1.11–1.21)	1.12 (1.07–1.16)	1.13 (1.08–1.19)	0.3028
Non-diabetic Women	1.45 (1.43–1.47)	1.47 (1.44–1.50)	1.56 (1.54–1.59)	1.51 (1.49–1.54)	1.53 (1.51–1.55)	<.0001
Diabetic Women	1.25 (1.19–1.30)	1.25 (1.21–1.29)	1.37 (1.30–1.44)	1.31 (1.22–1.39)	1.25 (1.20–1.30)	0.1507
Non- HDL-cholesterol (mmol/l)						
Non-diabetic Men	4.05 (4.01–4.10)	3.89 (3.84–3.94)	3.88 (3.84–3.92)	3.80 (3.76–3.84)	3.64 (3.59–3.69)	<.0001
Diabetic Men	3.96 (3.75–4.17)	4.01 (3.79–4.24)	4.05 (3.88–4.22)	3.63 (3.46–3.79)	3.60 (3.47–3.74)	<.0001
Non-diabetic Women	3.85 (3.81–3.90)	3.68 (3.63–3.74)	3.71 (3.66–3.76)	3.64 (3.60–3.68)	3.55 (3.51–3.59)	<.0001
Diabetic Women	4.26 (4.11–4.42)	4.07 (3.84–4.29)	3.95 (3.76–4.13)	3.79 (3.67–3.92)	3.67 (3.57–3.77)	<.0001
Triglycerides (mmol/l) ^d^						
Non-diabetic Men	1.52 (1.46–1.58)	1.45 (1.38–1.53)	1.36 (1.30–1.42)	1.38 (1.33–1.42)	1.41 (1.35–1.46)	0.0062
Diabetic Men	1.67 (1.54–1.81)	1.89 (1.64–2.13)	1.71 (1.52–1.89)	1.55 (1.31–1.79)	1.78 (1.58–1.99)	0.0292
Non-diabetic Women	1.32 (1.27–1.37)	1.31 (1.27–1.35)	1.22 (1.19–1.26)	1.23 (1.18–1.27)	1.22 (1.18–1.27)	0.0003
Diabetic Women	1.71 (1.54–1.88)	1.72 (1.48–1.96)	1.80 (1.47–2.14)	1.79 (1.56–2.03)	1.82 (1.65–2.00)	0.2096
LDL-cholesterol (mmol/l) ^d^						
Non-diabetic Men	3.41 (3.36–3.46)	3.28 (3.20–3.36)	3.04 (2.99–3.09)	3.04 (3.00–3.08)	2.97 (2.92–3.02)	<.0001
Diabetic Men	3.15 (2.91–3.38)	3.19 (2.87–3.51)	2.81 (2.61–3.01)	2.68 (2.45–2.91)	2.64 (2.46–2.82)	<.0001
Non-diabetic Women	3.24 (3.17–3.30)	3.13 (3.07–3.19)	2.98 (2.93–3.04)	3.00 (2.95–3.06)	2.97 (2.93–3.01)	<.0001
Diabetic Women	3.38 (3.23–3.52)	3.00 (2.83–3.17)	3.01 (2.76–3.26)	2.95 (2.70–3.20)	2.91 (2.75–3.08)	<.0001

^a^All estimates are weighted to be representative of the US noninstitutionalized population. Means were age-standardized by the direct method to the 2000 US Census population using the age categories of 20–39, 40–59 years, and ≥60 years

^b^Applying the Bonferroni method for adjusting for multiple comparisons for diabetic and non-diabetic men and women, there were 6 implied comparisons and an α = 0.008 (α = 0.05/6) was used

^c^Significant quadratic trend: *P* < 0.001 for diabetic women, adjusted for multiple comparisons

^d^Triglycerides, and LDL-cholesterol measurements were available only for persons examined in the morning session. Subsample fasting sample weights were used for triglycerides and LDL-cholesterol

The mean systolic BP level for diabetic women increased from 125 (95% CI, 123–127) mmHg in 1988–1994 to 130 (95% CI, 127–134) mmHg in 1999–2002 and then declined in 2011–2014 to 123 (95% CI, 122–125) mmHg (*P* = 0.014 for quadratic trend) (Table 1). For both men and women, the magnitude of declines was similar among diabetic and non-diabetic participants (for men, *P* for interactions between survey and diabetes status were 0.279 for systolic BP and 0. 053 for diastolic BP; for women, the corresponding figures were 0.322 and 0.471). In both the diabetic and non-diabetic state, absolute reductions in the mean systolic BP levels were comparable between men and women (*P* for interactions between survey and sex were 0.438 in the non-diabetic state and 0.101 in the diabetic state).

The age-adjusted mean TC, LDL-cholesterol, and non-HDL-cholesterol levels declined linearly among all diabetic and non-diabetic men and women groups (Table 1). For women, mean TC, LDL-cholesterol, and non-HDL-cholesterol levels tended to decline more over time among diabetic compared with non-diabetic persons (*P* for interactions between survey and diabetes status were all <0.001 for TC, LDL-cholesterol, and non-HDL-cholesterol). In the non-diabetic state, the survey × sex interaction terms comparing change in mean TC, LDL-cholesterol and non-HDL-cholesterol levels between the first and fifth surveys showed a statistically significantly greater reduction in men than in women (*P* for interactions between survey and sex were 0.0008 for TC, <0.001 for LDL-cholesterol, and 0.008 for non-HDL-cholesterol). In the diabetic state, men experienced similar magnitude of declines in mean TC, LDL-cholesterol and non-HDL-cholesterol levels as women (*P* for interactions between survey and sex were 0.964 for TC, 0.305 for LDL-cholesterol, and 0.958 for non-HDL-cholesterol).

There was not a significant trend in geometric mean triglycerides levels or mean HDL-cholesterol levels among diabetic patients. However, for both men and women, the extent of the changes in geometric mean triglycerides levels and mean HDL-cholesterol levels in diabetic patients were similar to those in non-diabetic persons (for men, *P* for interactions between survey and diabetes status were 0.439 for triglycerides, 0.04 for HDL-cholesterol; for women, the corresponding figures were 0.933 and 0.03, respectively). In both the diabetic and non-diabetic state, absolute changes in geometric mean triglycerides levels and mean HDL-cholesterol levels were comparable between men and women (in the non-diabetic state, *P* for interactions between survey and sex were 0.855 for triglycerides and 0.059 for HDL-cholesterol; in the diabetic state, the corresponding figures were 0.611 and 0.849, respectively).

For both men and women, there was a similar magnitude of decrease in the prevalence of smoking in diabetic and non-diabetic persons (for men and women, *P* for interactions between survey and diabetes status were 0.221 and 0.044, respectively) (Table 2). In both the diabetic and non-diabetic state, men experienced similar declines in rate of smoking as women (for diabetic and non-diabetic persons, *P* for interactions between survey and sex were 0.505 and 0.285, respectively).

**Table 2:** Age-adjusted prevalence of cardiovascular risk factors by sex and glucose-defined diabetes status among adults aged 20 years or older, 1988–2014^a^

Risk actors by sex and diabetes status	NHANES 1988–1994	NHANES 1999–2002	NHANES 2003–2006	NHANES 2007–2010	NHANES 2011–2014	*P* Value for Linear Trend, 1988–1994 to 2011-2014^b^	*P* Value for Comparison of NHANES 2011–2014 and NHANES 1988–1994
Smoking (%)							
Non-diabetic Men	32.52 (30.34–34.69)	22.78 (20.45–25.11)	23.68 (21.52–25.83)	20.54 (18.19–22.88)	19.01 (15.79–22.24)	<.0001	−13.51 (−17.39 to −9.62)
Diabetic Men	23.63 (17.33–29.92)	20.11 (14.89–25.32)	19.25 (14.15–24.35)	17.41 (14.25–20.56)	16.11 (10.12–22.11)	0.0218	−7.52 (−16.21 to 1.18)
Non-diabetic Women	25.19 (23.43–26.95)	18.85 (17.01–20.68)	18.51 (16.64–20.39)	16.16 (14.19–18.13)	13.55 (11.62–15.48)	<.0001	−11.64 (−14.25 to −9.03)
Diabetic Women	22.06 (17.82–26.29)	10.75 (6.86–14.64)	14.28 (8.92–19.64)	14.78 (11.26–18.29)	14.80 (7.71–21.89)	0.0081	−7.26 (−15.52 to 1.01)
Use of antihypertensive medications (%)							
Non-diabetic Men	9.04 (7.70–10.37)	15.11 (13.42–16.79)	19.84 (18.13–21.55)	20.57 (18.31–22.84)	21.13 (17.67–24.58)	<.0001	12.09 (8.38 to 15.80)
Diabetic Men	28.28 (23.20–33.36)	46.01 (39.71–52.31)	53.01 (48.03–58.00)	54.69 (49.57–59.81)	56.60 (47.16–66.03)	<.0001	28.32 (17.59 to 39.04)
Non-diabetic Women	12.38 (11.39–13.37)	19.60 (17.74–21.45)	21.72 (19.80–23.63)	23.15 (21.68–24.63)	23.80 (20.51–27.09)	<.0001	11.42 (7.98 to 14.86)
Diabetic Women	39.50 (34.03–44.97)	59.51 (53.51–65.51)	61.47 (56.51–66.43)	62.25 (56.90–67.61)	61.08 (55.65–66.52)	<.0001	21.58 (13.87 to 29.30)
Use of Lipid-Lowering Medications (%)							
Non-diabetic Men	2.16 (1.64–2.67)	8.38 (7.18–9.59)	10.97 (9.68–12.25)	13.32 (11.76–14.88)	13.33 (10.66–16.00)	<.0001	11.17 (8.45 to 13.89)
Diabetic Men	8.26 (4.73–11.79)	28.73 (24.50–32.96)	41.73 (35.53–47.92)	41.29 (37.25–45.33)	49.00 (41.86–56.14)	<.0001	40.74 (32.77 to 48.71)
Non-Diabetic Women	2.91 (2.21–3.61)	6.72 (5.79–7.64)	10.51 (8.88–12.13)	12.41 (11.17–13.64)	13.99 (10.94–17.05)	<.0001	11.08 (7.95 to 14.21)
Diabetic Women	6.92 (4.33–9.52)	26.26 (19.96–32.55)	36.28 (31.23–41.33)	38.38 (33.08–43.68)	49.68 (42.82–56.54)	<.0001	42.76 (35.42 to 50.09)
Prevalence of achieving systolic/diastolic blood pressure < 130/80 mmHg (%)							
Non-diabetic Men	71.93 (69.70–74.17)	72.67 (70.46–74.88)	71.43 (69.69–73.17)	76.32 (74.62–78.02)	77.22 (74.51–79.93)	<.0001	5.29 (1.77 to 8.80)
Diabetic Men	47.51 (40.52–54.50)	52.32 (46.82–57.81)	57.16 (50.81–63.51)	62.37 (57.68–67.07)	55.96 (46.33–65.60)	0.0019	8.45 (−3.46 to 20.37)
Non-diabetic Women	76.27 (74.31–78.22)	72.49 (70.71–74.26)	75.40 (73.73–77.07)	79.27 (77.63–80.91)	78.84 (76.54–81.15)	<.0001	2.57 (−0.44 to 5.60)
Diabetic Women	45.66 (40.96–50.36)	42.63 (35.39–49.87)	54.67 (49.25–60.09)	59.91 (55.73–64.08)	55.84 (50.98–60.69)	<.0001	10.18 (3.42 to 16.94)
Prevalence of achieving LDL-cholesterol <100 mg/dL (%)							
Non-diabetic Men	64.85 (62.73–66.98)	64.83 (62.60–67.07)	67.87 (66.17–69.57)	68.23 (66.46–69.99)	67.30 (63.50–71.10)	0.0005	2.45 (−1.90 to 6.81)
Diabetic Men	65.73 (59.80–71.66)	75.75 (70.28–81.22)	77.59 (73.40–81.77)	81.81 (78.17–85.46)	78.37 (73.59–83.16)	<.0001	12.64 (5.02 to 20.27)
Non-diabetic Women	66.90 (65.04–68.77)	67.42 (65.67–69.17)	70.55 (68.98–72.12)	67.76 (66.34–69.19)	67.04 (64.13–69.94)	0.0369	0.14 (−3.32 to 3.59)
Diabetic Women	65.36 (59.98–70.74)	79.47 (74.43–84.52)	76.74 (71.23–82.26)	80.83 (78.02–83.65)	76.65 (71.68–81.62)	<.0001	11.29 (3.96 to 18.62)
Prevalence of achieving HbA1c <7.0% (%)							
Diabetic Men	56.50 (50.01–62.99)	40.57 (34.30–46.84)	54.40 (49.59–59.22)	54.06 (48.05–60.07)	57.29 (51.38–63.20)	0.2121	0.79 (−8.00 to 9.58)
Diabetic Women	51.61 (44.90–58.33)	43.24 (36.66–49.83)	58.16 (52.93–63.39)	62.10 (57.22–66.99)	56.81 (49.80–63.81)	0.0002	5.20 (−4.51 to 14.91)

^a^All estimates are weighted to be representative of the US noninstitutionalized population. Means were age-standardized by the direct method to the 2000 US Census population using the age categories of 20–39, 40–59 years, and ≥60 years

^b^Applying the Bonferroni method for adjusting for multiple comparisons for diabetic and non-diabetic men and women, there were 6 implied comparisons and an α = 0.008 (α = 0.05/6) was used

There were increasing trends in the prevalence of antihypertensive and lipid-lowering medication use and the prevalence of achieving desirable systolic/diastolic BP and LDL-cholesterol levels among all diabetic and non-diabetic men and women groups (Table 2). An increasing trend in the prevalence of achieving desirable HbA1c levels were noted in diabetic women but not in diabetic men. For both men and women, the prevalence of antihypertensive and lipid-lowering medication use increased more among diabetic patients than among non-diabetic persons (for men, *P* for interactions between survey and diabetes status were 0.009 for antihypertensive medication use and <.0001 for lipid-lowering medication use; for women, *P* for interactions between survey and diabetes status were 0.001 for antihypertensive medication use and <.0001 for lipid-lowering medication use). For women, there was a greater magnitude of increase in the prevalence of achieving desirable systolic/diastolic BP and LDL-cholesterol levels among diabetic patients than among non-diabetic persons (*P* for interactions between survey and diabetes status were 0.0004 for achieving desirable systolic/diastolic BP levels and 0.009 for achieving desirable LDL-cholesterol levels). In both the diabetic and non-diabetic state, men experienced similar increments as women (in the non-diabetic state, *P* for interactions between survey and sex were 0.21 for antihypertensive medication use, 0.912 for lipid-lowering medication use, 0.744 for achieving desirable systolic/diastolic BP levels, and 0.06 for achieving desirable LDL-cholesterol levels; in the diabetic state, the corresponding figures were 0.18, 0.827, 0.426 and 0.505, respectively).

In 2011–2014, the sex × diabetes interaction became not statistically significant for all studied CVD risk factors except HDL-cholesterol (Table 1). In each survey, there was no statistical evidence for sex heterogeneity in the association of diabetes with the prevalence of smoking, patients taking antihypertensive and lipid-lowering medications, and persons with desirable systolic/diastolic BP and LDL-cholesterol levels (Table 2), suggesting that differences in these parameters between diabetic and non-diabetic persons did not differ by sex.

Results were remarkably similar when diabetes was defined by HbA1c ≥ 6.5% (48 mmol/mol), previously diagnosed diabetes, or current use of anti-diabetic medication or insulin (Tables 3 and 4), supporting recent guidelines recommending HbA1c as a diabetes diagnostic tool [14].

**Table 3:** Age-adjusted means of cardiovascular risk factors by sex and HbA1c-defined diabetes status among adults aged 20 years or older, 1988–2014^a^

Risk actors by sex and diabetes status	NHANES 1988–1994	NHANES 1999–2002	NHANES 2003–2006	NHANES 2007–2010	NHANES 2011–2014	*P* Value for Linear Trend, 1988–1994 to 2011-2014^b^
Body mass index (kg/m^2^)						
Non-diabetic Men	26.42 (26.21–26.63)	27.41 (27.19–27.63)	28.05 (27.81–28.28)	28.24 (27.98–28.5)	28.14 (27.91–28.37)	<.0001
Diabetic Men	29.61 (28.25–30.96)	33.02 (31.24–34.79)	32.40 (31.04–33.75)	32.55 (31.42–33.69)	33.49 (31.60–35.38)	<.0001
Non- diabetic Women	26.16 (25.86–26.46)	27.84 (27.48–28.20)	28.01 (27.60–28.42)	28.07 (27.82–28.32)	28.58 (28.22–28.94)	<.0001
Diabetic Women	31.97 (29.94–33.99)	33.48 (31.88–35.09)	35.04 (33.36–36.71)	36.45 (34.76–38.14)	35.84 (34.52–37.15)	<.0001
Waist circumference (cm)						
Non- diabetic Men	95.30 (94.89–95.70)	98.26 (97.66–98.87)	100.16 (99.50–100.82)	100.01 (99.28–100.75)	99.96 (99.32–100.59)	<.0001
Diabetic Men	104.35 (100.84–107.87)	113.04 (108.89–117.19)	110.93 (107.77–114.09)	110.78 (107.42–114.14)	114.61 (109.97–119.26)	<.0001
Non- diabetic Women	88.00 (87.31–88.68)	91.74 (90.83–92.64)	93.32 (92.40–94.24)	93.83 (93.16–94.50)	95.41 (94.61–96.21)	<.0001
Diabetic Women	102.38 (98.03–106.73)	106.57 (102.73–110.41)	109.25 (105.18–113.31)	114.15 (110.57–117.72)	112.37 (109.30–115.45)	<.0001
Systolic blood pressure (mmHg)						
Non-diabetic Men	125.16 (124.52–125.79)	124.40 (123.36–125.45)	124.23 (123.37–125.08)	122.77 (122.19–123.35)	122.53 (121.74–123.33)	<.0001
Diabetic Men	129.59 (126.81–132.37)	127.83 (124.63–131.04)	127.24 (124.22–130.25)	126.56 (124.38–128.75)	128.82 (126.41–131.23)	0.0008
Non- diabetic Women	120.16 (119.55–120.78)	122.23 (121.49–122.97)	120.68 (119.99–121.38)	118.17 (117.43–118.91)	118.73 (118.02–119.43)	<.0001
Diabetic Women	126.89 (124.55–129.24)	130.50 (127.19–133.81)	127.97 (125.70–130.24)	125.50 (123.39–127.61)	124.35 (122.04–126.67)^c^	<.0001
Diastolic blood pressure (mmHg)						
Non-diabetic Men	76.70 (76.27–77.12)	73.86 (73.19–74.54)	71.68 (71.12–72.24)	71.98 (71.26–72.70)	71.77 (70.94–72.60)	<.0001
Diabetic Men	77.88 (75.91–79.85)	75.80 (72.40–79.20)	73.79 (71.22–76.37)	70.88 (68.41–73.34)	73.37 (70.89–75.86)	<.0001
Non-diabetic Women	72.26 (71.82–72.70)	70.87 (70.17–71.58)	69.31 (68.68–69.95)	68.42 (67.55–69.29)	69.58 (68.94–70.22)	<.0001
Diabetic Women	74.63 (73.40–75.86)	70.03 (66.85–73.21)	69.73 (67.79–71.67)	70.01 (68.25–71.76)	70.69 (69.25–72.13)	<.0001
Total cholesterol (mmol/l)						
Non-diabetic Men	5.24 (5.19–5.29)	5.09 (5.04–5.14)	5.15 (5.11–5.18)	5.03 (4.98–5.08)	4.90 (4.86–4.95)	<.0001
Diabetic Men	5.24 (4.88–5.59)	5.06 (4.84–5.29)	5.21 (5.00–5.42)	4.78 (4.61–4.95)	4.69 (4.50–4.89)	<.0001
Non-diabetic Women	5.30 (5.26–5.35)	5.15 (5.10–5.20)	5.28 (5.24–5.32)	5.15 (5.11–5.19)	5.08 (5.04–5.11)	<.0001
Diabetic Women	5.52 (5.36–5.68)	5.31 (5.12–5.50)	5.20 (4.97–5.44)	5.20 (4.96–5.43)	4.95 (4.82–5.07)	<.0001
HDL-cholesterol (mmol/l)						
Non-diabetic Men	1.2 (1.18–1.22)	1.21 (1.19–1.22)	1.27 (1.26–1.29)	1.23 (1.21–1.25)	1.26 (1.24–1.28)	<.0001
Diabetic Men	1.11 (1.03–1.19)	1.05 (0.98–1.11)	1.15 (1.08–1.21)	1.13 (1.07–1.18)	1.12 (1.07–1.16)	0.0388
Non-diabetic Women	1.45 (1.42–1.47)	1.47 (1.44–1.5)	1.56 (1.54–1.59)	1.51 (1.49–1.54)	1.53 (1.51–1.55)	<.0001
Diabetic Women	1.25 (1.19–1.31)	1.25 (1.21–1.3)	1.36 (1.28–1.43)	1.27 (1.19–1.34)	1.22 (1.18–1.26)	0.8459
Non- HDL-cholesterol (mmol/l)						
Non-diabetic Men	4.04 (3.99–4.09)	3.89 (3.84–3.94)	3.88 (3.84–3.92)	3.8 (3.75–3.84)	3.64 (3.6–3.69)	<.0001
Diabetic Men	4.12 (3.69–4.55)	4.01 (3.79–4.23)	4.06 (3.85–4.27)	3.65 (3.46–3.84)	3.58 (3.37–3.79)	<.0001
Non-diabetic Women	3.86 (3.81–3.91)	3.68 (3.63–3.74)	3.72 (3.67–3.77)	3.64 (3.6–3.68)	3.55 (3.51–3.59)	<.0001
Diabetic Women	4.27 (4.09–4.45)	4.06 (3.85–4.27)	3.85 (3.63–4.06)	3.93 (3.72–4.14)	3.72 (3.6–3.85)	<.0001
Triglycerides (mmol/l) ^d^						
Non-diabetic Men	1.52 (1.45–1.58)	1.45 (1.37–1.53)	1.36 (1.31–1.42)	1.38 (1.33–1.42)	1.41 (1.36–1.46)	0.0123
Diabetic Men	1.93 (1.67–2.19)	1.87 (1.66–2.07)	1.67 (1.49–1.86)	1.57 (1.32–1.82)	1.74 (1.51–1.98)	0.0034
Non-diabetic Women	1.33 (1.28–1.37)	1.32 (1.28–1.36)	1.23 (1.19–1.26)	1.23 (1.18–1.27)	1.22 (1.18–1.27)	<.0001
Diabetic Women	1.81 (1.76–1.86)	1.64 (1.39–1.9)	1.78 (1.43–2.13)	1.79 (1.55–2.04)	1.83 (1.66–2.01)	0.2453
LDL-cholesterol (mmol/l) ^d^						
Non-diabetic Men	3.39 (3.34–3.44)	3.28 (3.2–3.35)	3.04 (2.98–3.09)	3.04 (3–3.09)	2.97 (2.91–3.02)	<.0001
Diabetic Men	3.45 (3.25–3.66)	3.13 (2.86–3.4)	2.81 (2.6–3.02)	2.68 (2.48–2.89)	2.58 (2.35–2.81)	<.0001
Non-diabetic Women	3.24 (3.18–3.31)	3.13 (3.07–3.18)	2.98 (2.93–3.04)	3 (2.95–3.06)	2.97 (2.93–3.02)	<.0001
Diabetic Women	3.31 (3.23–3.39)	3.02 (2.87–3.17)	2.99 (2.73–3.25)	2.99 (2.72–3.26)	2.9 (2.74–3.06)	<.0001

^a^All estimates are weighted to be representative of the US noninstitutionalized population. Means were age-standardized by the direct method to the 2000 US Census population using the age categories of 20–39, 40–59 years, and ≥60 years

^b^Applying the Bonferroni method for adjusting for multiple comparisons for diabetic and non-diabetic men and women, there were 6 implied comparisons and an α = 0.008 (α = 0.05/6) was used

^c^Significant quadratic trend: *P* < 0.001 for diabetic women, adjusted for multiple comparisons

^d^Triglycerides, and LDL-cholesterol measurements were available only for persons examined in the morning session. Subsample fasting sample weights were used for triglycerides and LDL-cholesterol

**Table 4:** Age-adjusted prevalence of cardiovascular risk factors by sex and HbA1c-defined diabetes status among adults aged 20 years or older, 1988–2014^a^

Risk actors by sex and diabetes status	NHANES 1988–1994	NHANES 1999–2002	NHANES 2003–2006	NHANES 2007–2010	NHANES 2011–2014	*P* Value for Linear Trend, 1988–1994 to 2011-2014^b^	*P* Value for Comparison of NHANES 2011–2014 and NHANES 1988–1994
Smoking (%)							
Non-diabetic Men	32.64 (30.48–34.80)	22.96 (20.64–25.27)	23.63 (21.42–25.83)	20.35 (18.10–22.61)	18.74 (15.86–21.63)	<.0001	−13.90 (−17.50 to −10.30)
Diabetic Men	20.48 (15.50–25.46)	20.08 (15.12–25.04)	18.89 (13.38–24.41)	18.76 (14.97–22.55)	18.23 (10.70–25.75)	0.1560	−2.25 (−11.29 to 6.78)
Non-diabetic Women	25.32 (23.55–27.09)	18.88 (16.96–20.81)	18.86 (16.90–20.83)	16.25 (14.34–18.16)	13.92 (11.95–15.90)	<.0001	−11.40 (−14.05 to −8.75)
Diabetic Women	19.44 (14.95–23.93)	9.57 (6.33–12.81)	11.00 (6.85–15.15)	14.11 (10.27–17.94)	13.21 (7.83–18.59)	0.0501	−6.23 (−13.25 to 0.78)
Use of antihypertensive medications (%)							
Non-diabetic Men	9.03 (7.74–10.32)	15.16 (13.48–16.84)	20.02 (18.28–21.76)	21.07 (18.81–23.32)	20.98 (17.59–24.38)	<.0001	11.95 (8.33 to 15.58)
Diabetic Men	31.32 (25.48–37.16)	45.67 (39.25–52.08)	55.49 (51.12–59.86)	54.54 (49.08–60.00)	62.19 (53.98–70.39)	<.0001	30.87 (20.79 to 40.95)
Non-diabetic Women	12.45 (11.50–13.40)	19.87 (17.93–21.82)	21.64 (19.74–23.55)	23.09 (21.56–24.63)	23.90 (20.51–27.28)	<.0001	11.45 (7.93 to 14.96)
Diabetic Women	41.68 (36.37–46.99)	59.86 (53.44–66.28)	65.60 (60.13–71.08)	70.62 (66.68–74.57)	64.53 (59.03–70.03)	<.0001	22.85 (15.20 to 30.50)
Use of Lipid-Lowering Medications (%)							
Non-diabetic Men	2.13 (1.62–2.64)	8.38 (7.16–9.61)	11.20 (9.91–12.50)	13.24 (11.70–14.79)	14.00 (11.12–16.88)	<.0001	11.87 (8.94 to 14.79)
Diabetic Men	9.56 (5.86–13.27)	28.33 (24.28–32.39)	42.83 (36.78–48.88)	45.50 (42.02–48.98)	49.15 (41.97–56.33)	<.0001	39.59 (31.50 to 47.67)
Non-diabetic Women	2.97 (2.28–3.66)	6.69 (5.75–7.63)	10.32 (8.75–11.89)	12.29 (11.10–13.47)	14.14 (11.19–17.10)	<.0001	11.17 (8.14 to 14.21)
Diabetic Women	6.43 (4.01–8.86)	26.12 (19.86–32.38)	40.79 (34.63–46.94)	45.02 (39.76–50.28)	51.72 (43.41–60.03)	<.0001	45.29 (36.63 to 53.95)
Prevalence of achieving systolic/diastolic blood pressure < 130/80 mmHg (%)							
Non-diabetic Men	72.00 (69.80–74.20)	72.80 (70.59–75.01)	71.45 (69.66–73.25)	76.39 (74.61–78.17)	76.88 (73.96–79.80)	<.0001	4.88 (1.22 to 8.54)
Diabetic Men	42.84 (35.58–50.10)	52.33 (47.70–56.96)	55.27 (47.10–63.43)	59.69 (55.58–63.81)	55.22 (45.02–65.42)	0.0018	12.38 (−0.15 to 24.91)
Non-diabetic Women	76.39 (74.34–78.44)	72.04 (70.19–73.90)	75.85 (74.16–77.55)	79.15 (77.42–80.88)	78.49 (76.36–80.62)	<.0001	2.10 (−0.85 to 5.06)
Diabetic Women	39.87 (34.57–45.18)	41.98 (35.16–48.81)	46.74 (41.14–52.34)	57.19 (53.18–61.20)	54.12 (47.65–60.58)	<.0001	14.25 (5.88 to 22.61)
Prevalence of achieving LDL-cholesterol <100 mg/dL (%)							
Non-diabetic Men	64.69 (62.55–66.83)	64.99 (62.72–67.27)	68.02 (66.23–69.81)	68.82 (67.10–70.54)	68.08 (64.45–71.71)	<.0001	3.39 (−0.82 to 7.60)
Diabetic Men	68.23 (62.30–74.16)	77.81 (71.93–83.69)	78.18 (73.69–82.68)	79.29 (75.22–83.36)	76.98 (70.72–83.25)	0.0014	8.75 (0.12 to 17.39)
Non-diabetic Women	66.42 (64.60–68.24)	68.06 (66.25–69.87)	70.97 (69.28–72.65)	68.87 (67.41–70.34)	68.01 (64.84–71.18)	0.0016	1.59 (−2.07 to 5.24)
Diabetic Women	73.26 (67.97–78.55)	79.64 (74.13–85.14)	76.82 (70.86–82.78)	75.59 (71.87–79.32)	75.47 (70.81–80.13)	0.6815	2.21 (−4.85 to 9.26)
Prevalence of achieving HbA1c <7.0% (%)							
Diabetic Men	42.28 (36.06–48.50)	38.10 (33.12–43.08)	43.52 (38.05–48.99)	43.42 (38.21–48.63)	50.58 (43.53–57.63)	0.1042	8.30 (−1.11 to 17.70)
Diabetic Women	35.97 (29.91–42.02)	38.56 (33.29–43.84)	50.23 (44.08–56.39)	49.31 (44.73–53.89)	50.83 (44.25–57.40)	<.0001	14.86 (5.91 to 23.80)

^a^All estimates are weighted to be representative of the US noninstitutionalized population. Means were age-standardized by the direct method to the 2000 US Census population using the age categories of 20–39, 40–59 years, and ≥60 years

^b^Applying the Bonferroni method for adjusting for multiple comparisons for diabetic and non-diabetic men and women, there were 6 implied comparisons and an α = 0.008 (α = 0.05/6) was used

## Discussion

In this series of nationally representative surveys, reductions in some of the major CVD risk factors and increments in intervention measures benefited men and women with and without diabetes, which mirror the changes in the general US population [16, 17]. Diabetic men experienced similar declines in mean systolic/diastolic BP, TC, triglycerides, HDL-cholesterol, rate of smoking and similar increments in rates of achieving desirable systolic/diastolic BP and LDL-cholesterol levels compared with non-diabetic men. Diabetic women experienced a greater reduction in mean TC, LDL-cholesterol and non-HDL-cholesterol, and a greater increase in rates of achieving desirable systolic/diastolic BP and LDL-cholesterol levels compared with non-diabetic women. Thus, diabetic men and women may be at lower CVD risk now than in previous eras. All of the improvements were observed approximately equally in diabetic men and diabetic women, indicating that the greater relative risk of CVD in diabetic women compared with diabetic men [18, 19] may be dissipated now.

Several factors could explain the favorable trends in systolic/diastolic BP and lipid levels, ranging from healthy lifestyle changes to pharmacological factors [20–22]. Smoking rate has declined [22] and more patients use antihypertensive and lipid-lowering medications, which was observed in our study.

Previous NHANES study compared CVD risk factors between 1971 and 1974 and 1999–2000 [23]. Our earlier time period used data from 1988 to 1994, and our later time period contained data collected as recently as 2014. The period between 1988 and 1994 and 1999–2000 has seen impressive evidence regarding the benefits of control of BP and lipid levels [24–26], which may contribute to statistically significant decrease in systolic/diastolic BP and lipid levels in individuals with diabetes. Hence, our present report allows a much longer period over which to detect improvements in CVD risk factors. Landmark studies have repeatedly shown the importance of reduction of cholesterol and BP in reducing CVD mortality among diabetic patients [27, 28]. Several trials of statin use among diabetic patients have shown that lipid lowering is associated with a reduction in CVD events [28]. Compelling evidence demonstrates that BP control can dramatically delay or prevent the microvascular and macrovascular complications of diabetes [27]. In addition, BP control has been reported to be the most cost-effective intervention [29].

Our findings that the magnitude of reductions in mean TC, LDL-cholesterol, and non-HDL-cholesterol, and the rates of persons achieving desirable systolic/diastolic BP and LDL-cholesterol levels among diabetic women exceeded those among non-diabetic women challenge the viewpoint that diabetic women receive less medical management and the presence of diabetes in women reduces the benefit of improved medical treatments [30]. Our present findings occurred in the setting of an unprecedented body of evidence from clinical trials demonstrating significant benefits of BP and lipid control in those with diabetes [24–26]. An interesting and promising finding may be that diabetic women may have comparable or greater improvement in CVD survival compared with non-diabetic women. Actually, recent studies illustrated that diabetic women experience a greater improvement in CVD survival than non-diabetic women [2].

Our finding that diabetic women had as good an improvement in CVD risk factors as diabetic men challenge another viewpoint that diabetes having an absolute greater detrimental effect in women [5, 31]. Accumulating data reported a greater difference in CVD risk factors between diabetic and nondiabetic women than between diabetic and nondiabetic men. However, in 2011–2014, we observed no statistical evidence for sex heterogeneity in the association of diabetes with the major CVD risk factors. A possible explanation for these discrepancies is that the sex homogeneity is a recent phenomenon. Our finding that the differences in CVD risk profile between diabetic and nondiabetic women is similar to that between diabetic and nondiabetic men supports recent studies showing the similarly favorable reductions in CVD mortality in men and women with diabetes [2].

The favorable changes in the major CVD risk factors and increased lipid-lowering and antihypertensive medication use among adults with diabetes observed in our study suggest that the health care and management of those with diabetes has genuinely improved in recent years. Despite the strong scientific evidence showing the benefits of the aggressive promotion of BP and lipid control in reducing CVD mortality among persons with diabetes [29, 32], many patients do not reach the treatment targets for BP and lipids [33, 34]. Hence, ongoing efforts remain necessary to promote CVD risk factor reduction.

The prevalence of achieving desirable HbA1c levels decreased in diabetic men and women between 1988 and 1994 and 1999–2002, it increased thereafter. Explanations for the quadratic trends in the prevalence of achieving desirable HbA1c levels remain to be elucidated. Almost half of U.S. adults with diabetes did not achieve HbA1c goals. Given the strong scientific evidence showing the benefits of achieving desirable HbA1c levels [35] in reducing CVD mortality, highlighting the need for further improvements in glycemic control is important.

BMI and WC were notable exceptions to the observed reduction in risk factors, as mean BMI and WC levels increased among all diabetic and non-diabetic men and women. The importance of obesity as a major CVD risk factor has received considerable attention [8]. Increased BMI and WC may be increasing the burden of CVD caused by diabetes, highlighting the need for further improvements in weight control.

There are several limitations to this study. First, the NHANES surveys are cross-sectional in design, and, thus, we cannot directly draw the causes of changes in CVD risk factors. Second, since time point represents data from a different cross-sectional sample, differential sampling error may affect comparisons over time. Third, the sample is nationally representative of U.S. adults, and therefore, extrapolating results to other populations should be interpreted cautiously.

## Conclusions

In conclusion, between 1988 and 2014, there were favorable trends in reductions of the major CVD risk factors and increments in intervention measures among men and women with and without diabetes. The major CVD risk factors in men with diabetes decreased to the same extent that they did for men without diabetes. The magnitude of changes in the favorable trends in diabetic women was of similar or greater compared with those among non-diabetic women. Diabetic men and diabetic women equally share all of the studied improvements.

## Additional file

Additional file 1: Table S1. Survey Sample Characteristics. (DOCX 25 kb)

## Abbreviations

ADA: American Diabetes Association
CVD: Cardiovascular disease
FPG: Fasting plasma glucose
HbA1c: Hemoglobin A1c
NHANES: National Health and Nutrition Examination Surveys
TC: Total cholesterol
WC: Waist circumference
