# Supplementary material for: Trends in cardiovascular risk factors among U.S. men and women with and without diabetes, 1988–2014
Source: BMC Public Health. 2017 Nov 22;17:893. doi: 10.1186/s12889-017-4921-4 (PMC5700686; doi:10.1186/s12889-017-4921-4)
Supplement: Additional file 1: Table S1. — Survey Sample Characteristics. (DOCX 25 kb) [file 12889_2017_4921_MOESM1_ESM.docx]

Supplemental Table 1. Survey Sample Characteristics^a^

| Risk actors by diabetes status | | NHANES 1988-1994 | NHANES 1999-2002 | NHANES 2003-2006 | NHANES 2007-2010 | NHANES 2011-2014 |
| --- | --- | --- | --- | --- | --- | --- |
| Female (%） | Non-diabetes | 52.19 (51.28-53.09) | 52.17 (51.30-53.05) | 52.33 (51.39-53.27) | 52.09 (51.11-53.07) | 52.71 (50.94-54.47) |
|  | Diabetes | 47.58 (43.49-51.67) | 48.98 (45.10-52.86) | 50.51 (47.97-53.05) | 50.00 (46.95-53.06) | 47.31 (42.71-51.91) |
| Non-Hispanic black (%) | Diabetes | 10.22 (8.96-11.48) | 9.86 (7.68-12.04) | 10.75 (8.15-13.35) | 10.26 (8.36-12.16) | 10.26 (6.16-14.37) |
|  | Non-diabetes | 15.17 (12.4-17.94) | 17.66 (12.44-22.89) | 15.98 (12.40-19.57) | 13.75 (10.58-16.92) | 16.05 (9.26-22.84) |
| Age (years) | Non-diabetes | 46.00 (45.77-46.22) | 46.26 (46.04-46.48) | 46.20 (46.00-46.40) | 46.11 (45.94-46.27) | 45.96 (45.76-46.17) |
|  | Diabetes | 47.36 (46.54-48.18) | 48.19 (47.69-48.70) | 48.14 (47.59-48.69) | 47.97 (47.40-48.53) | 47.55 (47.04-48.07) |
| Body mass index (kg/m^2^) | Non-diabetes | 26.30 (26.09-26.51) | 27.66 (27.39-27.92) | 28.03 (27.76-28.30) | 28.18 (27.99-28.36) | 28.36 (28.10-28.63) |
|  | Diabetes | 29.35 (28.54-30.16) | 32.93 (31.38-34.48) | 33.23 (32.22-34.23) | 32.98 (31.95-34.02) | 33.80 (32.94-34.65) |
| Waist circumference (cm) | Non-diabetes | 91.47 (91.04-91.89) | 94.87 (94.24-95.50) | 96.55 (95.86-97.24) | 96.82 (96.28-97.36) | 97.53 (96.93-98.12) |
|  | Diabetes | 100.34 (98.7-101.98) | 108.90 (105.26-112.54) | 109.13 (106.96-111.31) | 109.13 (106.92-111.34) | 111.42 (109.23-113.61) |
| Systolic blood pressure (mmHg) | Non-diabetes | 122.72 (122.25-123.19) | 123.38 (122.60-124.16) | 122.58 (121.90-123.26) | 120.47 (119.95-120.98) | 120.63 (119.94-121.31) |
|  | Diabetes | 126.97 (125.30-128.63) | 129.30 (126.43-132.17) | 127.01 (125.20-128.81) | 125.37 (123.62-127.12) | 124.94 (123.81-126.07) |
| Diastolic blood pressure (mmHg) | Non-diabetes | 74.45 (74.07-74.82) | 72.33 (71.73-72.93) | 70.51 (70.01-71.01) | 70.15 (69.35-70.95) | 70.73 (70.05-71.40) |
|  | Diabetes | 75.01 (74.15-75.87) | 72.45 (69.69-75.21) | 71.54 (69.85-73.23) | 69.98 (68.51-71.46) | 71.25 (69.93-72.58) |
| Total cholesterol (mmol/l) | Non-diabetes | 5.29 (5.25-5.32) | 5.13 (5.09-5.18) | 5.22 (5.19-5.25) | 5.10 (5.07-5.14) | 5.00 (4.97-5.03) |
|  | Diabetes | 5.29 (5.16-5.42) | 5.20 (5.03-5.37) | 5.26 (5.12-5.41) | 4.92 (4.81-5.03) | 4.82 (4.74-4.90) |
| HDL-cholesterol (mmol/l) | Non-diabetes | 1.33 (1.31-1.35) | 1.34 (1.32-1.36) | 1.42 (1.41-1.44) | 1.38 (1.36-1.40) | 1.40 (1.38-1.42) |
|  | Diabetes | 1.19 (1.16-1.23) | 1.16 (1.12-1.20) | 1.26 (1.21-1.31) | 1.21 (1.16-1.26) | 1.19 (1.15-1.22) |
| Non- HDL-cholesterol (mmol/l) | Non-diabetes | 3.96 (3.91-4.00) | 3.79 (3.74-3.83) | 3.79 (3.76-3.83) | 3.72 (3.70-3.75) | 3.60 (3.56-3.63) |
|  | Diabetes | 4.09 (3.95-4.24) | 4.04 (3.85-4.22) | 4.01 (3.87-4.14) | 3.71 (3.61-3.81) | 3.63 (3.54-3.72) |
| Triglycerides (mmol/l)^b^ | Non-diabetes | 1.42 (1.37-1.46) | 1.38 (1.33-1.43) | 1.29 (1.25-1.32) | 1.30 (1.27-1.34) | 1.31 (1.27-1.35) |
|  | Diabetes | 1.69 (1.58-1.8) | 1.80 (1.64-1.96) | 1.75 (1.54-1.96) | 1.66 (1.49-1.84) | 1.79 (1.65-1.93) |
| LDL-cholesterol (mmol/l) ^b^ | Non-diabetes | 3.32 (3.28-3.37) | 3.21 (3.15-3.26) | 3.01 (2.97-3.06) | 3.03 (2.99-3.07) | 2.98 (2.94-3.01) |
|  | Diabetes | 3.25 (3.11-3.39) | 3.10 (2.93-3.26) | 2.90 (2.74-3.06) | 2.81 (2.66-2.97) | 2.80 (2.67-2.92) |
| Smoking (%) | Non-diabetes | 28.69 (26.92-30.47) | 20.73 (18.92-22.53) | 20.97 (19.17-22.78) | 18.25 (16.30-20.21) | 16.13 (14.17-18.09) |
|  | Diabetes | 22.88 (18.66-27.09) | 15.52 (12.38-18.67) | 16.74 (13.46-20.02) | 16.09 (14.14-18.04) | 15.49 (13.24-17.74) |
| Use of antihypertensive medications (%) | Non-diabetes | 10.78 (9.8-11.77) | 17.45 (15.91-18.99) | 20.82 (19.30-22.35) | 21.92 (20.34-23.50) | 22.53 (19.41-25.66) |
|  | Diabetes | 33.62 (30.18-37.06) | 52.62 (47.62-57.63) | 57.28 (54.03-60.54) | 58.47 (55.03-61.92) | 58.72 (53.21-64.23) |
| Use of Lipid-Lowering Medications (%) | Non-diabetes | 2.55 (2.07-3.03) | 7.51 (6.61-8.41) | 10.73 (9.51-11.94) | 12.84 (11.75-13.94) | 13.68 (11.35-16.00) |
|  | Diabetes | 7.62 (5.53-9.72) | 27.52 (23.70-31.34) | 38.98 (35.31-42.64) | 39.84 (37.03-42.64) | 49.32 (45.09-53.55) |
| Prevalence of achieving systolic/diastolic blood pressure <130/80 mmHg (%) | Non-diabetes | 74.19 (72.46-75.93) | 72.57 (70.87-74.27) | 73.51 (72.14-74.87) | 77.86 (76.59-79.12) | 78.08 (76.01-80.14) |
|  | Diabetes | 46.63 (42.28-50.98) | 47.57 (42.67-52.47) | 55.90 (51.36-60.45) | 61.14 (57.72-64.56) | 55.90 (50.84-60.97) |
| Prevalence of achieving LDL-cholesterol <100 mg/dL achieving (%) | Non-diabetes | 65.92 (64.31-67.54) | 66.18 (64.57-67.80) | 69.27 (68.06-70.49) | 67.98 (66.58-69.39) | 67.16 (64.39-69.94) |
|  | Diabetes | 65.55 (61.1-70.01) | 77.57 (73.34-81.81) | 77.16 (73.90-80.42) | 81.32 (79.02-83.63) | 77.56 (73.62-81.50) |

^a^Estimates are weighted to be representative of the US noninstitutionalized population aged ≥ 20 years.

^b^ Triglycerides, and LDL-cholesterol measurements were available only for persons examined in the morning session. Subsample fasting sample weights were used for triglycerides and LDL-cholesterol.
